# Nosocomial infections in in-hospital cardiac arrest patients who undergo extracorporeal cardiopulmonary resuscitation

**DOI:** 10.1371/journal.pone.0243838

**Published:** 2020-12-23

**Authors:** Ryoung-Eun Ko, Kyungmin Huh, Dong-Hoon Kim, Soo Jin Na, Chi Ryang Chung, Yang Hyun Cho, Kyeongman Jeon, Gee Young Suh, Jeong Hoon Yang

**Affiliations:** 1 Department of Critical Care Medicine, Samsung Medical Center, Sungkyunkwan University School of Medicine, Seoul, Republic of Korea; 2 Division of Infectious Diseases, Department of Medicine, Samsung Medical Center, Sungkyunkwan University School of Medicine, Seoul, Republic of Korea; 3 Department of Thoracic and Cardiovascular Surgery, Samsung Medical Center, Sungkyunkwan University School of Medicine, Seoul, Republic of Korea; 4 Division of Pulmonary and Critical Care Medicine, Department of Medicine, Samsung Medical Center, Sungkyunkwan University School of Medicine, Seoul, Korea; 5 Division of Cardiology, Department of Medicine, Samsung Medical Center, Sungkyunkwan University School of Medicine, Seoul, Republic of Korea; St. Michael's Hospital, CANADA

## Abstract

**Background:**

Little is known of nosocomial infections (NI) in patients who suffer from in-hospital cardiac arrest who undergoing extracorporeal cardiopulmonary resuscitation. This study aimed to investigate clinical pictures of NI, and the association of NIs with clinical outcomes in in-hospital cardiac arrest patients who undergoing extracorporeal cardiopulmonary resuscitation.

**Methods:**

To evaluate the incidence and clinical characteristics of NI in patients who undergoing extracorporeal cardiopulmonary resuscitation, a retrospective cohort study was conducted in a single tertiary referral center between January 2010 and December 2018. We included adult patients who undergoing extracorporeal cardiopulmonary resuscitation for in-hospital cardiac arrest and excluded patients who were out-of-hospital cardiac arrest or failed ECMO implantation. Clinical characteristics and outcomes were compared between NI and Non-NI patients, or multidrug-resistant (MDR) and non-MDR. The independent risk factors associated with NIs were also analyzed using multivariable logistic regression model.

**Results:**

Thirty-five (23.3%) patients developed a NI. These cases included 21 patients with a gram negative (G-) infection, 12 patients with a gram positive (G+) bacterial infection, and two patients with fungal infection. Pneumonia was the most common type of NIs, followed by catheter-related infection. The in-hospital mortality and neurologic outcomes at discharge were not different between the NI and non-NI groups. Multidrug-resistant (MDR) pathogens were detected in 10 cases (28.6%). The MDR NI patients had a higher ICU mortality than did those with non-MDR NI (80% vs. 32%, p = 0.028). Following multivariable adjustment, body mass index (adjusted OR 0.87, 95% CI, 0.77–0.97, p = 0.016) and cardiopulmonary resuscitation to pump on time (adjusted OR 1.04, 95% CI, 1.01–1.06, p = 0.001) were independent predictors of NI development.

**Conclusions:**

In patients who received extracorporeal cardiopulmonary resuscitation, NIs were not associated with an increase in in-hospital mortality. However, NIs with MDR organisms do increase the risk of in-hospital mortality. Lower body mass index and longer low flow time were significant predictors of NI development.

## Introduction

Extracorporeal cardiopulmonary resuscitation (ECPR) for refractory cardiac arrest involves the use of veno-arterial extracorporeal membrane oxygenation (ECMO) in addition to standard resuscitative efforts [1]. Rapid cannulation plays a crucial role in reducing the hypoxic brain injury, because it can reduce the low flow time during ECPR [2]. However, in emergent ECPR practice, it is very difficult to perform a clean procedure quickly. In addition, the indwelling catheters that are used for veno-arterial ECMO can also be risk factors for nosocomial infection (NI) development [3]. Traditionally, NI remains a major cause of morbidity and mortality in intensive care unit (ICU). The risk of developing NIs might be inevitably increased in ECPR patients with immunocompromised conditions and who have additional indwelling medical devices such as central lines, arterial lines, renal replacement therapy, and invasive mechanical ventilation [4–6]. However, to date, only a few studies have evaluated the incidence, risk factors, microbial etiology, and antibiotic resistance patterns of NIs in patients with acute respiratory distress syndrome require prolonged support of veno-venous ECMO [7–9]. There are no data regarding the clinical features and outcomes of NIs in ECPR patients. Therefore, we sought to investigate the incidence, microbial etiology, resistance patterns, risk factors of NIs, and the association between NIs and clinical outcomes in in-hospital cardiac arrest (IHCA) patients who underwent ECPR.

## Materials and methods

### Study population

This is a retrospective, single-center, observational study of adult patients who underwent ECPR for IHCA between January 2010 and December 2018. This study was approved by the Institutional Review Board of Samsung Medical Center (No. 2019-10-119). The requirement for informed consent was waived given the study’s retrospective nature. The clinical and laboratory data were collected by a trained study coordinator using a standardized case report form. All consecutive patients older than 18 years who underwent ECPR were screened for study inclusion. Patients who received ECPR due to out-of-hospital cardiac arrest or failed ECMO implantation were excluded.

### Standard care

Protocol-based approaches to infection control and prevention are applied in the ICUs of Samsung Medical Center. The following ventilator-associated pneumonia prevention bundles are used: 1) elevation head of the bed; 2) stress ulcer prevention; 3) pain assessment and sedation scale evaluation every 8 hours. Early enteral feeding is recommended. However, prophylactic or selective decontamination antibiotic regimens are not employed. Catheters and cannulas insertion sites are monitored daily, and transparent dressings are applied routinely. We use needle-free closed systems for drug infusion and blood withdrawal. There is no scheduled indwelling catheter removal. Infection control measures are monitored. Alcohol-based hand hygiene is implemented. Strict individual contact precautions and patient cohort isolation apply from the time the patient is admitted to the exclusion of colonization and/or infection by multidrug-resistant (MDR) bacteria. At the time of admission, patients also undergo surveillance perineal swabs for Vancomycin-resistant *Enterococci* and Carbapenem-resistant *Enterobacteriaceae*. In contrast, no routine tracheal, blood, or urine cultures are performed. We manage septic patients according to internationally accepted guidelines. We do not use an antibiotic prescription protocol. Instead, we use computerized protocol and follow the Infection Disease Society of America Guidelines for diagnosis and treatment of NIs [10,11]. In particular, the antibiotic regimens are revised daily by a dedicated infectious diseases specialist and clinical pharmacist after communication with the microbiology laboratory [12].

#### Definition and outcomes

ECPR was defined as successful veno-arterial ECMO implantation and pump-on with chest compression for external cardiac massage during the index procedure in patients with cardiac arrest. The resuscitation procedure was performed in the same way as described in our previous study [13,14]. Cases in which ECPR was deferred included a short life expectancy (< 6 months), terminal malignancy, an unwitnessed collapse, limited physical activity, an unprotected airway, or those in which CPR had already been performed for more than 60 minutes at the time of the initial contact. Age alone was not a contraindication to ECPR. When a return of spontaneous circulation is achieved during ECMO cannulation, the practitioners typically do not remove the inserted cannula or stop the ECMO pump-on process. ECMO pump-on is defined by the status in which chest compression were stopped following successful ECMO implantation and activation. At this time, the ECMO flow was gradually increased until a patient’s respiratory and hemodynamic statuses stabilized. The CPR to ECMO pump-on time was defined by that from the initiation of chest compressions to the time at which the ECMO pump was turned on.

We retrospectively evaluated all of the positive microbiological cultures that were obtained between 24 hours after the beginning of ECMO support until 48 hours after decannulation. These data were obtained based on the available clinical, laboratory, and radiographic data following international guidelines [15–17]. The following NIs were diagnosed: pneumonias, catheter-associated urinary tract infections, bloodstream infections, and catheter-related blood stream infections (CRBSI) (S1 Table). We only included each patient’s first episode of NI in this study. ECMO cannula insertion site infection was diagnosed when all of the following were present: 1) local erythema and purulent drainage; and 2) positive cultures of the purulent drainage for microorganisms other than common skin contaminants [7]. The MDR pathogens were defined according to the Center for Disease Control definition [18].

We assessed the incidence and clinical characteristics of NIs and compared clinical outcomes between NI and Non-NI patients, or MDR and non-MDR. Clinical outcomes included ICU length of stay, ICU mortality, hospital mortality, ECMO related complication, and Cerebral Performance Category score at hospital discharge. In addition, we evaluated the independent risk factors associated with development of NIs.

#### Statistical analysis

Data are presented as medians and interquartile ranges (IQR) for continuous variables, and as numbers (percentages) for categorical variables. The baseline characteristics and outcomes measures of interest were compared among the NI and non-NI groups. The Mann-Whitney U test was used to compare continuous variables, while the chi-square test or Fisher’s exact test was used for categorical variables. All of the tests were two-sided, and P values <0.05 were considered statistically significant. We performed a multivariable logistic regression analysis after adjusting for age, malignancy, and factors with p < 0.2 on univariate analysis in order to estimate whether the factor was associated with NI during ECMO. All of the data analyses were performed using R Statistical Software. (Version 3.2.5; R Foundation for Statistical Computing, Vienna, Austria).

## Results

### Study population

**Fig 1:**
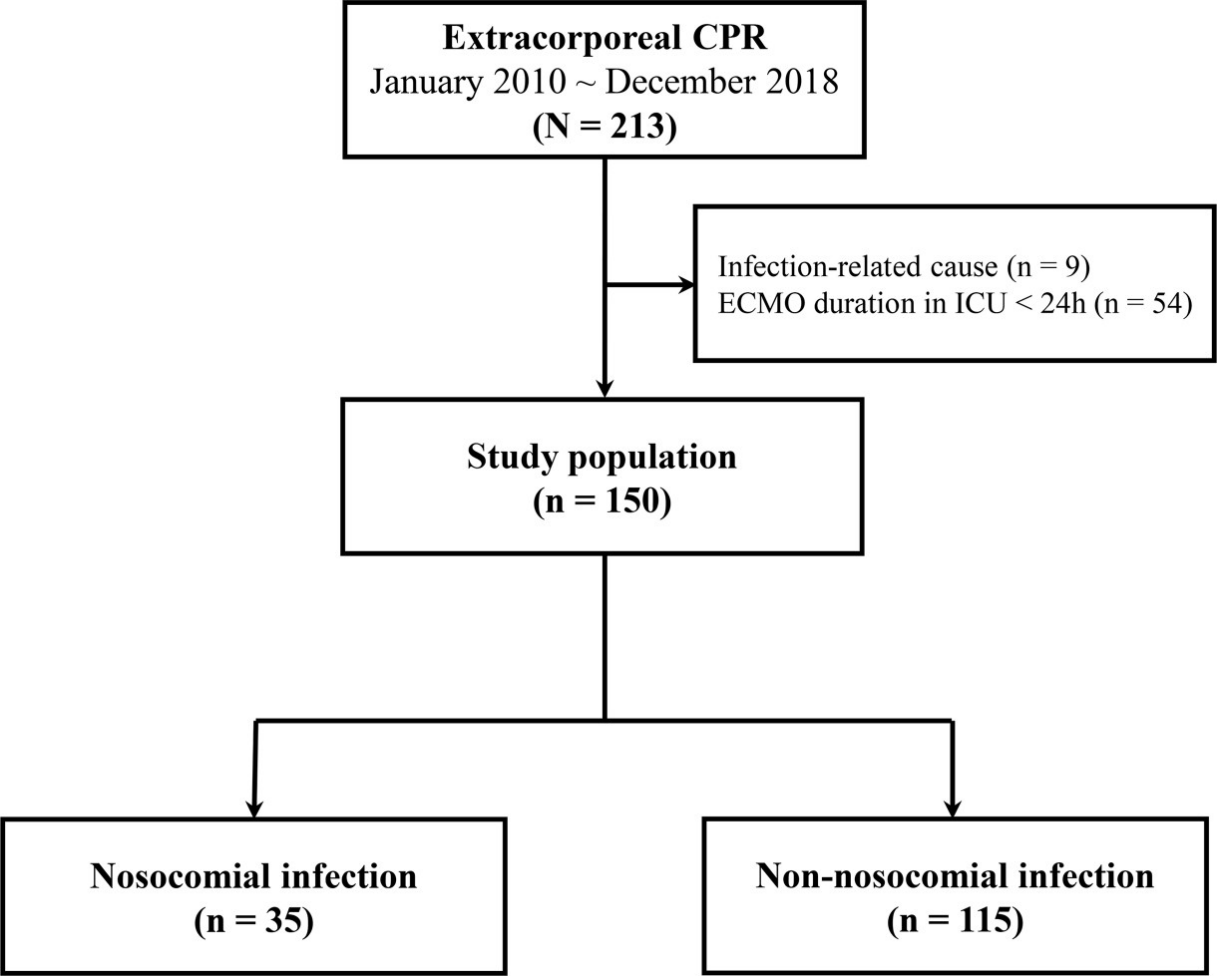
Study flow chart. CPR = cardiopulmonary resuscitation, ECMO = extracorporeal membrane oxygenation, ICU = intensive care unit.

Between January 2010 and December 2018, 213 patients underwent ECPR. We ultimately analyzed 150 patients who maintained ECMO for more than 24 hours (Fig 1). The median patient age was 60 (IQR 51.0–72.0) years, and 74.7% of the patients were male. Ninety six (64%) patients underwent ECPR within 2 days after hospital admission. Detailed characteristics of the resuscitation was described according to Utstein template in S2 Table. Of these patients, 35 (23.3%) developed a NI during their ECMO course, while 115 (76.7%) did not. The incidence rate of the first NI was 1.7 infections per 1,000 ECMO hours. The patients’ characteristics, comorbidities, and laboratory data on the day of ECPR are summarized in Table 1. There were no differences between the two groups with the exception of the history of percutaneous coronary intervention and lower body mass index (BMI) in the NI group.

**Table 1:** Clinical characteristics and initial management.

	Infection (n = 35)	Non-infection (n = 115)	p
Age (year)	67.0 [52.5–73.0]	60.0 [50.0–69.5]	0.277
Sex, male	23 (65.7)	89 (77.4)	0.242
BMI (kg/m^2^)	22.3 [21.0–24.3]	24.5 [22.1–27.4]	0.012
Medical history			
Diabetes mellitus	14 (40.0)	50 (43.5)	0.866
Hypertension	18 (51.4)	54 (47.0)	0.787
Malignancy	7 (20.0)	11 (9.6)	0.172
Dyslipidemia	5 (14.3)	17 (14.8)	1.000
Chronic kidney disease^a^	5 (14.3)	15 (13.0)	1.000
Previous myocardial infarction	7 (20.0)	34 (29.6)	0.371
Cerebral vascular disease	2 (5.7)	13 (11.3)	0.520
History of PCI	4 (11.4)	37 (32.2)	0.028
History of CABG	3 (8.6)	6 (5.2)	0.745
History of heart transplantation	2 (5.7)	3 (2.6)	0.720
Laboratory data on the day of ECPR			
Initial lactate (mmol/L)	8.7 [3.3–14.0]	8.1 [3.6–11.8]	0.589
Serum glucose maximum (mg/dL)	314.0 [275.5–435.0]	305.0 [248.0–369.0]	0.217
Hemoglobin before ECMO (g/dL)	10.8 [9.2–13.2]	11.8 [9.9–14.5]	0.238
Total bilirubin (mg/dL)	1.0 [0.6–2.0]	0.9 [0.5–1.4]	0.100
Creatinine (mg/dL)	1.4 [1.2–1.9]	1.3 [1.0–2.0]	0.395
ANC (x10^3^/μL)	8.3 [5.8–12.6]	7.4 [4.4–11.7]	0.395
C-reactive protein (mg/dL)	1.0 [0.1–4.2]	0.6 [0.1–6.0]	0.582
Procalcitonin (ng/mL)	0.6 [0.3–1.9]	0.5 [0.2–6.2]	0.946
Hospitalization prior to ECPR, day	6.6 ± 8.6	8.2 ± 39.1	0.673
Cause of arrest			0.613
Ischemic	16 (45.7)	69 (60.0)	
Dilated cardiomyopathy	1 (2.9)	3 (2.6)	
Myocarditis	0 (0.0)	1 (0.9)	
Stress-induced cardiomyopathy	3 (8.6)	2 (1.7)	
Rejection after heart transplantation	1 (2.9)	3 (2.6)	
Valvular heart disease	2 (5.7)	4 (3.5)	
Acute aortic syndrome	1 (2.9)	3 (2.6)	
Pulmonary thromboembolism	3 (8.6)	9 (7.8)	
Refractory arrhythmia	5 (14.3)	12 (10.4)	
Hypovolemic shock	2 (5.7)	6 (5.2)	
Others^b^	1 (2.9)	3 (2.6)	
Initial rhythm			0.316
Asystole	7 (20.0)	13 (11.3)	
Pulseless electrical activity	16 (45.7)	50 (43.5)	
Shockable rhythm	12 (34.3)	52 (45.2)	
CPR to pump-on time (min)	36.0 [26.5–48.5]	28.0 [20.0–40.0]	0.030
ROSC before ECMO	17 (48.6)	48 (41.7)	0.603
Location of CPR			0.913
Intensive care unit	15 (42.9)	41 (35.7)	
Catheterization laboratory	6 (17.1)	26 (22.6)	
Operation room	2 (5.7)	5 (4.3)	
Emergency room	10 (28.6)	37 (32.2)	
General ward	2 (5.7)	6 (5.2)	
Location of insertion			0.868
Intensive care unit	14 (40.0)	44 (38.3)	
Catheterization laboratory	8 (22.9)	33 (28.7)	
Operation room	2 (5.7)	4 (3.5)	
Emergency room	11 (31.4)	34 (29.6)	
Percutaneous insertion	34 (97.1)	113 (99.1)	0.960
Distal perfusion	15 (42.9)	46 (40.4)	0.946
Targeted temperature management	7 (20.0)	26 (22.6)	0.945
Initial post ECPR management			
Prophylactic antibiotics	29 (82.9)	95 (82.6)	1.000
Mechanical ventilation	29 (82.9)	93 (80.9)	0.987
Renal replacement therapy	21 (60.0)	58 (50.4)	0.424
Vasopressor	31 (93.9)	102 (97.1)	0.745
ECMO duration (hour)	122.0 [58.5–200.5]	71.0 [45.0–122.5]	0.006

Presented values are medians with interquartile ranges in parentheses, mean ± standard deviation or numbers with percentages in parentheses.

^a^Chronic kidney disease is defined as either kidney damage or GFR <60mL/min/1.73 m^2^ for ≥ 3 months.

^b^Others include 3 pulmonary hypertension patients and one post pneumonectomy syndrome patient.

BMI = body mass index, PCI = percutaneous coronary intervention, CABG = coronary artery bypass grafting, ECMO = extracorporeal membrane oxygenation, ANC = absolute neutrophil count, CPR = cardiopulmonary resuscitation, ROSC = return of spontaneous circulation, ECPR = extracorporeal cardiopulmonary resuscitation.

#### Procedural characteristics

The characteristics of cardiac arrest and initial managements are also described in Table 1. Eighty-five (56.7%) were arrested due to ischemic causes and 65 (43.3%) had a return of spontaneous circulation before ECMO pump-on. The procedural characteristics during ECPR were similar between the two groups. However, the CPR to pump-on time was significantly longer in the NI group than it was in the non-NI group (36 minutes vs. 28 minutes, p < 0.03). Peripheral cannulation was performed using the Seldinger technique in most patients (98.7%). On the day of ECPR, 124 (82.7%) patients were treated with prophylactic antibiotics. Mechanical ventilation was used in 122 (81.3%) patients, renal replacement therapy was started in 79 (52.7%) patients, and vasopressors were required in 133 (96.4%) patients. The ECMO duration was significantly longer in the NI group than it was in the non-NI group (122 hours vs. 71 hours, p *=* 0.006).

#### Nosocomial infection

The type of NI, and the causative microorganisms are listed in S2 Table. There were 21 patients with Gram negative (G-) infections, 12 patients with Gram positive (G+) infections, and two patients with fungal infections. Pneumonia was the most common type of NI, followed by CRBSI. Of 19 patients with pneumonia, 3 patients was diagnosed hospital-acquired pneumonia and 16 patients were diagnosed ventilator acquired pneumonia. CRBSIs with bacteria developed in 9 (6.0%) patients, all of whom were infected with the *Staphylococcus* species. There were two CRBSIs with Candida species. Seven (4.7%) patients were diagnosed with a primary bacteremia and one (0.7%) was diagnosed with a urinary tract infection. MDR pathogens were detected in 10 cases (28.6%). Most NIs (57.1%) developed within 3 days of ECPR (S1 Fig). Compared with non-MDR NIs, the MDR NIs were more often G+ infections (20.0% vs. 70.0%, p = 0.015) and more often CRBSIs (12.0% vs. 60.0%, p = 0.012).

Seven patients were diagnosed with a cannula site infection after the ECMO cannula was removed. Gram negative bacteria were common pathogens of ECMO cannula site infections (85.7%, *Klebsiella pneumoniae* 2 patients, *Klebsiella oxytoca* 2 patients, *Enterobacter cloacae* 1 patient, *Pseudomonas aeruginosa* 1 patient, and *Staphylococcus epidermidis* 1 patient). The incidence rate of ECMO cannula site infection was 9.0 infections/1,000 ECMO days. The detailed information for seven patients is described in S3 Table.

#### Clinical outcomes

**Fig 2:**
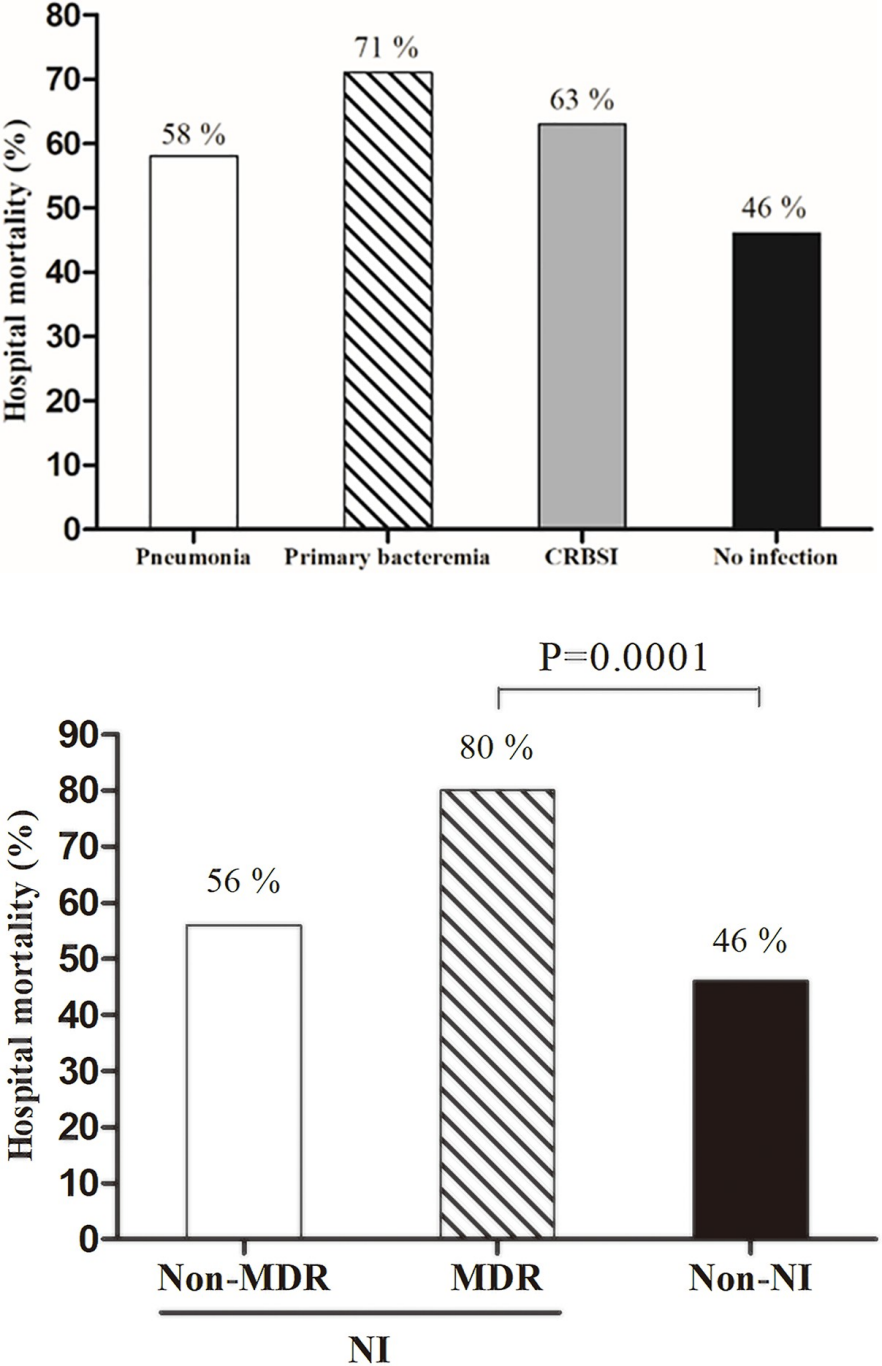
Mortality outcomes among IHCA patients. (A) According to nosocomial infection type, (B) According to resistance pattern. CRBSI, catheter-related bloodstream infection; MDR, multidrug resistant; NI, nosocomial infection.

The two groups did not differ with regard to in-hospital mortality, ICU mortality, or neurologic outcomes (Table 2). In addition, hospital mortality did not differ according to the NI type (Fig 2A). The ICU length of stay was significantly longer in the NI group than it was in the non-NI group (13 days vs. 8 days, p = 0.043). There was a significantly higher incidence of MDR infections in patients who developed NI after 3 days of ECMO initiation than there was in patients who developed NI within 3 days of ECMO initiation (46.6% vs. 20.0%, p < 0.001). The clinical characteristics and outcomes, according to the presence or absence of MDR infection, are presented in Table 3. Compared the patients with non-MDR infections, those with MDR infections had significantly higher in ICU mortality (32% vs. 80%, p = 0.028) and in-hospital mortality (46% vs. 82%, p < 0.001, Fig 2B).

**Table 2:** Clinical outcomes according to the presence of nosocomial infection.

	Infection (n = 35)	Non-infection (n = 115)	p
ICU length of stay (day)	12.8 [5.8–23.3]	8.0 [4.0–18.7]	0.043
ICU mortality	16 (45.7)	44 (38.3)	0.554
Hospital mortality	22 (62.9)	53 (46.1)	0.209
ECMO related complication			
Limb ischemia	4 (11.4)	10 (8.7)	0.877
Groin hematoma	0 (0.0)	5 (7.0)	0.506
ECMO site bleeding	4 (11.4)	6 (5.2)	0.367
Stroke	4 (11.4)	6 (5.2)	0.367
Femoral dissection	0 (0.0)	1 (1.4)	1.000
CPC score			0.912
1	15 (42.9)	41 (35.7)	
2	6 (17.1)	26 (22.6)	
3	2 (5.7)	5 (4.3)	
4	10 (28.6)	35 (30.4)	
5	2 (5.7)	6 (5.2)	

Presented values are medians with interquartile ranges in parentheses or numbers with percentages in parentheses.

ECMO = extracorporeal membrane oxygenation, ICU = intensive care unit, CPC = Cerebral Performance Category.

**Table 3:** Characteristics and outcomes according to the presence of MDR in patients with nosocomial infection.

	Non-MDR (n = 25)	MDR (n = 10)	p
Characteristics			
G +	5 (20.0)	7 (70.0)	0.015
G -	18 (72.0)	3 (30.0)	0.056
Pneumonia	14 (56.0)	5 (50.0)	1.000
Urinary tract infection	1 (4.0)	0 (0.0)	1.000
Primary bacteremia	7 (28.0)	0 (0.0)	0.161
Catheter related bloodstream infection	3 (12.0)	6 (60.0)	0.012
Outcomes			
ICU length of stay (day)	11.0 [5.2–20.4]	16.8 [11.0–30.9]	0.116
ICU mortality	8 (32.0)	8 (80.0)	0.028
Hospital mortality	14 (56.0)	8 (80.0)	0.347
ECMO length of stay (day)	3.9 [2.5–5.9]	6.9 [2.2–18.3]	0.546
CPC score			
1	5 (20.0)	0 (0.0)	
2	4 (16.0)	1 (10.0)	
3	1 (4.0)	1 (10.0)	
4	2 (8.0)	1 (10.0)	
5	13 (52.0)	7 (70.0)	

Presented values are medians with interquartile ranges in parentheses or numbers with percentages in parentheses.

ECMO = extracorporeal membrane oxygenation, MDR = multidrug-resistant, ICU = intensive care unit, CPC = Cerebral Performance Category.

#### Factors associated with increased nosocomial infections

On univariable analysis, the following parameters were predictors of NI development: BMI, CPR to pump on time, ECMO duration, and underlying malignancy. The following multivariable adjustments were independent predictors for the development of NIs: BMI (adjusted OR 0.87, 95% CI, 0.77–0.97, p = 0.016), and CPR to pump on time (adjusted OR 1.04, 95% CI, 1.01–1.06, p = 0.001).

## Discussion

In this study, we investigated the incidence, microbial etiology, risk factors, and impact of NI on the clinical outcomes of IHCA patients who underwent ECPR. The major findings of this study were as follows: (1) A substantial portion of ECPR patients suffered from NIs including pneumonias, catheter-associated urinary tract infections, primary bacteremia, and CRBSIs; (2) The NIs did not increase in-hospital mortality in IHCA patients undergoing ECPR; (3) However, MDR infections increased ICU mortality compared to that with non-MDR infections; (4) NI development was associated with lower BMI and longer CPR to pump on time.

NIs increase the risk of morbidity and mortality in hospitalized patients. The prevalence of NIs in the ICU has increased significantly with the increasing use of invasive devices in critically ill patients [19]. In particular, ECPR patients are more susceptible to infection given the need for various invasive procedures, such as a large size indwelling cannula for ECMO. Previous studies have reported a wide range of NI rates, from 21.4% to 64.0%, in patients who received ECMO [5,7,8,20]. The Extracorporeal Life Support Organization, which tracks international ECMO data, has reported NI rates in adults of 20.5% [21]. Although the NI rates of 23.3% in our study with short maintenance periods of ECMO was similar to that of previous studies, it seems relatively high because previous studies evaluated NIs in various registries including a substantial portion of veno-venous ECMO patients with prolonged ECMO duration [7,20,21]. Therefore, ECPR patients must be carefully monitored for NI development regardless of maintenance duration of ECMO.

Previous studies in veno-venous ECMO reported the incidence of CBSRI in VV ECMO up to 35% and ECMO device-related blood stream infections up to 6.8% [22,23]. In our study, the CBSRI were observed 9% of patients This result might be associated with the duration of ECMO in ECPR (75 [IQR 48–140] hours) is shorter than that of veno-venous ECMO in our previous study (14 [IQR 7–26] days) [9,24].

In this study, MDR infection was an important risk factor of ICU mortality in NI patients. These MDR infections are associated with serious mortality and increased cost in the ICU [25,26]. The MDR pathogens can typically survive in an environment where several antimicrobials are used. Patients in the ICU are at particular risk of, MDR infections, where long term combination antibiotic therapies are frequently used. In our analysis, the MDR pathogens occurred more frequently in NIs along with prolonged ECMO duration. Therefore, in this situation, physicians must carefully consider the possibility of MDR infection when NI occurs in prolonged maintenance after ECPR.

Previous studies have shown that age, autoimmune comorbidities, higher Sequential Organ Failure Assessment score, and ECMO duration are predictors of NI during ECMO [5,9,21,27]. Contrast to previous studies, we found that decreased BMI and longer CPR to pump on time were associated with NIs in IHCA patients undergoing ECPR. CPR to pump on time is one of the important modifiable prognostic factors of ECPR outcomes [2]. Furthermore, longer CPR to pump on time might be associated with less sterile procedures because CPR time depends on how quickly physician can do cannulation and the higher severity of illness at ECMO initiation. However, given that ECPR is a labor-intensive procedure with limited resources, it is difficult to reduce the CPR to pump on time without a well-organized ECMO team [24,28]. Accordingly, a well-organized ECMO team is required not only to improve clinical outcomes but also to reduce the incidence of infections. In this study, lower BMI was a significant predictor of NI. The study population consisted of nine underweight (6.0%), 77 normal weight (51.3%), 50 overweight (33.3%), and 14 obese (9.3%) patients. Although the mechanism regarding the relationship between low BMI and NIs is unclear, we can suspect that lower BMI may occur in those patients with underlying chronic diseases or malnutrition vulnerable to infection. The present result is consistent with previous research [29,30].

This study has several limitations. First, given its retrospective and single-center observational nature, it was subject to selection bias that may have influenced our findings. Therefore, well-designed prospective study is needed to confirm our results. Post hoc power analysis revealed that the current sample size would provide 62% statistical power as one-sided test. A second limitation is that this study was conducted over a long period of time, over which ICU management has changed. Therefore, differences in ICU patient management may have affected patient outcomes during the study period. However NI rate per year in ECPR were not different (S2 Fig). Third, we only analyzed microbiologically confirmed infections. The clinical diagnosis of infection in ECMO patients is challenging, because such patients invariably have signs of systemic inflammatory responses. This response may be triggered by the ECMO itself. In addition, fever is often non-apparent in ECMO patients, because body temperature is controlled by extracorporeal circulation. Fourth, it might be difficult to identify the onset of infection in some patients with positive culture that develops early after ECPR. Finally, despite prophylactic antibiotic were not routinely used in standard care of ICU, prophylactic use of antibiotic has increased due to suspected aspiration pneumonia or concerns about sterile procedure during the ECPR.

## Conclusions

In IHCA patients who underwent ECPR, NIs were not associated with increased risk of in-hospital mortality, although MDR infections did increase in-hospital mortality. Lower BMI and longer CPR to pump on time were significant predictors of NI development in ECPR patients.

## Supporting information

S1 Table Diagnostic criteria for infections. (DOCX)

S2 Table Characteristics of the resuscitation according to Utstein template. (DOCX)

S3 Table Microorganisms of first nosocomial infection (n = 35). (DOCX)

S4 Table Patients who diagnosed cannula site infection after the ECMO cannula. (DOCX)

S1 Fig Frequency of nosocomial infection and MDR infection. MDR = multidrug resistant, ECPR = extracorporeal cardiopulmonary resuscitation.
(TIF)

S2 Fig (TIF)
