# Supplementary material for: Nosocomial infections in in-hospital cardiac arrest patients who undergo extracorporeal cardiopulmonary resuscitation
Source: PLoS One. 2020 Dec 23;15(12):e0243838. doi: 10.1371/journal.pone.0243838 (PMC7757900; doi:10.1371/journal.pone.0243838)
Supplement: S1 Table — (DOCX) [file pone.0243838.s001.docx]

**S1 Table**. **Diagnostic criteria for infections.**

| Infection | Site of Culture | Bacterial load | Clinical signs | Also |
| --- | --- | --- | --- | --- |
| Pneumonia | Endotracheal aspirate | ≥ 10^6^ CFU/ml | Two of : fever, leukocytosis/leucopenia, purulent secretions + New/progressive radiographic infiltrate  + Worsening oxygenation | |
| Catheter-associated urinary tract infection | 2 consecutive urine specimens | ≥ 10^5^ CFU/ml | Fever/tachycardia/hypotension | |
| Primary bacteremia | 2 percutaneous blood samples  +  eventual blood from catheters |  | Fever/tachycardia/hypotension  +  No further sign of localized infection | No differential time to positivity between percutaneous and catheters |
| Catheter-related bloodstream infection | 2 percutaneous  +  catheter blood  or  catheter tip |  | Fever/tachycardia/hypotension  +  No further sign of localized infection. Eventual erythema, swelling, purulent drainage from catheter insertion-site. | Differential time to positivity > 2 hours  or  catheter CFU > 3-fold percutaneous CFU  or  positive catheter tip |
| ECMO cannula insertion site infection |  |  | Skin and soft tissue infection at the ECMO cannulation site. | |

CFU = colony forming units, ECMO = extracorporeal membrane oxygenation.
