# Supplementary material for: Nosocomial infections in in-hospital cardiac arrest patients who undergo extracorporeal cardiopulmonary resuscitation
Source: PLoS One. 2020 Dec 23;15(12):e0243838. doi: 10.1371/journal.pone.0243838 (PMC7757900; doi:10.1371/journal.pone.0243838)
Supplement: S2 Table — (DOCX) [file pone.0243838.s002.docx]

**S2 Table. The characteristics of the resuscitation according to Utstein template**

| Hospital^*^ |  |
| --- | --- |
| Number of hospital admissions, per year (over 18 years) | 91,668 |
| Number of treated in-hospital cardiac arrest, per year | 92 |
| Patient |  |
| Age | 60.0 [51.0 – 72.0] |
| Sex, male | 112 (74.7) |
| Pre-event |  |
| Patient category |  |
| Inpatient | 150 (100.0) |
| Illness category |  |
| Medical | 140 (93.3) |
| Surgical | 10 (6.7) |
| Cardiac arrest process |  |
| Location |  |
| Intensive care unit | 56 (37.3) |
| Catheterization laboratory | 32 (21.3) |
| Operation room | 7 (4.7) |
| Emergency room | 47 (31.3) |
| General ward | 8 (5.3) |
| Witnessed | 150 (100.0) |
| Initial rhythm |  |
| Asystole | 20 (13.3) |
| Pulseless electrical activity | 66 (44.0) |
| Shockable rhythm | 64 (42.7) |
| Number of shock delivered | 4.0 [2.0 – 7.0] |
| Epinephrine given | 38 (25.3) |
| Other drug given | 42 (28.0) |
| Airway interventions | 78 (52.0) |
| Post-resuscitation process |  |
| Targeted temperature management | 33 (22.0) |
| Coronary angiography | 58 (38.7) |
| Outcome |  |
| Survival to discharge | 75 (50.0) |
| Good neurologic prognosis | 88 (58.7) |

^*^Data has been obtained at 2018.
