# Supplementary material for: Nosocomial infections in in-hospital cardiac arrest patients who undergo extracorporeal cardiopulmonary resuscitation
Source: PLoS One. 2020 Dec 23;15(12):e0243838. doi: 10.1371/journal.pone.0243838 (PMC7757900; doi:10.1371/journal.pone.0243838)
Supplement: S3 Table — (DOCX) [file pone.0243838.s003.docx]

**S3 Table**. **Microorganisms of first nosocomial infection (n = 35).**

| Gram staining | Microorganism | Pneumonia | Urinary tract infection | Primary bacteremia | Catheter related bloodstream infection | Overall | MDR |
| --- | --- | --- | --- | --- | --- | --- | --- |
| Number (% of the included patients) | | 19 (12.7) | 1 (0.7) | 7 (4.7) | 9 (6.0) | 35 | 10 |
| G- |  | 13 (68.4) | 1 (100.0) | 5 (71.4) | 2 (22.2) | 21 | 3 |
|  | *Enterobacter aerogenes* | 5 | - | 2 | - | 7 |  |
|  | *Enterobacter cloacae* | 2 | - | 1 | - | 3 |  |
|  | *Klebsiella pneumonia* | - | - | 1 | 1 | 2 | 1 |
|  | *Acinetobacter baumanii* | 2 | - | - | 1 | 3 | 2 |
|  | *Pseudomonas spp.* | 1 | - | - | - | 1 |  |
|  | *Stenotrophomonas maltophilia* | 1 | - | - | - | 1 |  |
|  | *Bacteroides fragilis* | 1 | - | - | - | 1 |  |
|  | *Escherichia coli* | 1 | 1 | - | - | 2 |  |
|  | *Raoultella planticola* | - | - | 1 | - | 1 |  |
| G+ |  | 6 (31.6) | 0 (0.0) | 2 (28.6) | 5 (55.6) | 12 | 7 |
|  | *Staphylococcus aureus* | 5 | - | - | - | 5 | 3 |
|  | *Coagulase-negative Staphylococci* | - | - | 1 | 4 | 5 | 2 |
|  | *Enterococcus spp.* | 1 | - | - | 1 | 1 | 1 |
|  | *Streptococcus spp.* |  | - | 1 | - | 1 | 1 |
| Fungal |  | - | - | - | 2 | 2 | - |

Presented values are medians with interquartile ranges in parentheses or numbers with percentages in parentheses.
