# Supplementary material for: Nosocomial infections in in-hospital cardiac arrest patients who undergo extracorporeal cardiopulmonary resuscitation
Source: PLoS One. 2020 Dec 23;15(12):e0243838. doi: 10.1371/journal.pone.0243838 (PMC7757900; doi:10.1371/journal.pone.0243838)
Supplement: S4 Table — (DOCX) [file pone.0243838.s004.docx]

**S4 Table**. **Patients who diagnosed cannula site infection after the ECMO cannula.**

| Patients No. | Age | Gender | BMI | Insertion location | CPR to pump on time (min) | ECMO duration (day) | ICU mortality | Hospital  mortality |
| --- | --- | --- | --- | --- | --- | --- | --- | --- |
| 1 | 61 | Female | 23.6 | ICU | 47 | 1.9 | Survived | Survived |
| 2 | 50 | Male | 22.6 | ICU | 17 | 6.8 | Survived | Survived |
| 3 | 75 | Male | 21.4 | ER | 20 | 5.2 | Survived | Died |
| 4 | 25 | Male | 25.3 | ICU | 20 | 1.9 | Survived | Survived |
| 5 | 70 | Male | 24.3 | ICU | 32 | 2.6 | Died | Died |
| 6 | 27 | Male | 26.7 | ER | 53 | 2.0 | Survived | Survived |
| 7 | 61 | Male | 25.7 | Cath lab | 16 | 5.4 | Survived | Survived |

ECMO = extracorporeal membrane oxygenation, BMI = body mass index, ICU = intensive care unit, ER = emergency room, CPR = cardiopulmonary resuscitation.
