# Supplementary figures and images for: Nosocomial infections in in-hospital cardiac arrest patients who undergo extracorporeal cardiopulmonary resuscitation
Source: PLoS One. 2020 Dec 23;15(12):e0243838. doi: 10.1371/journal.pone.0243838 (PMC7757900; doi:10.1371/journal.pone.0243838)

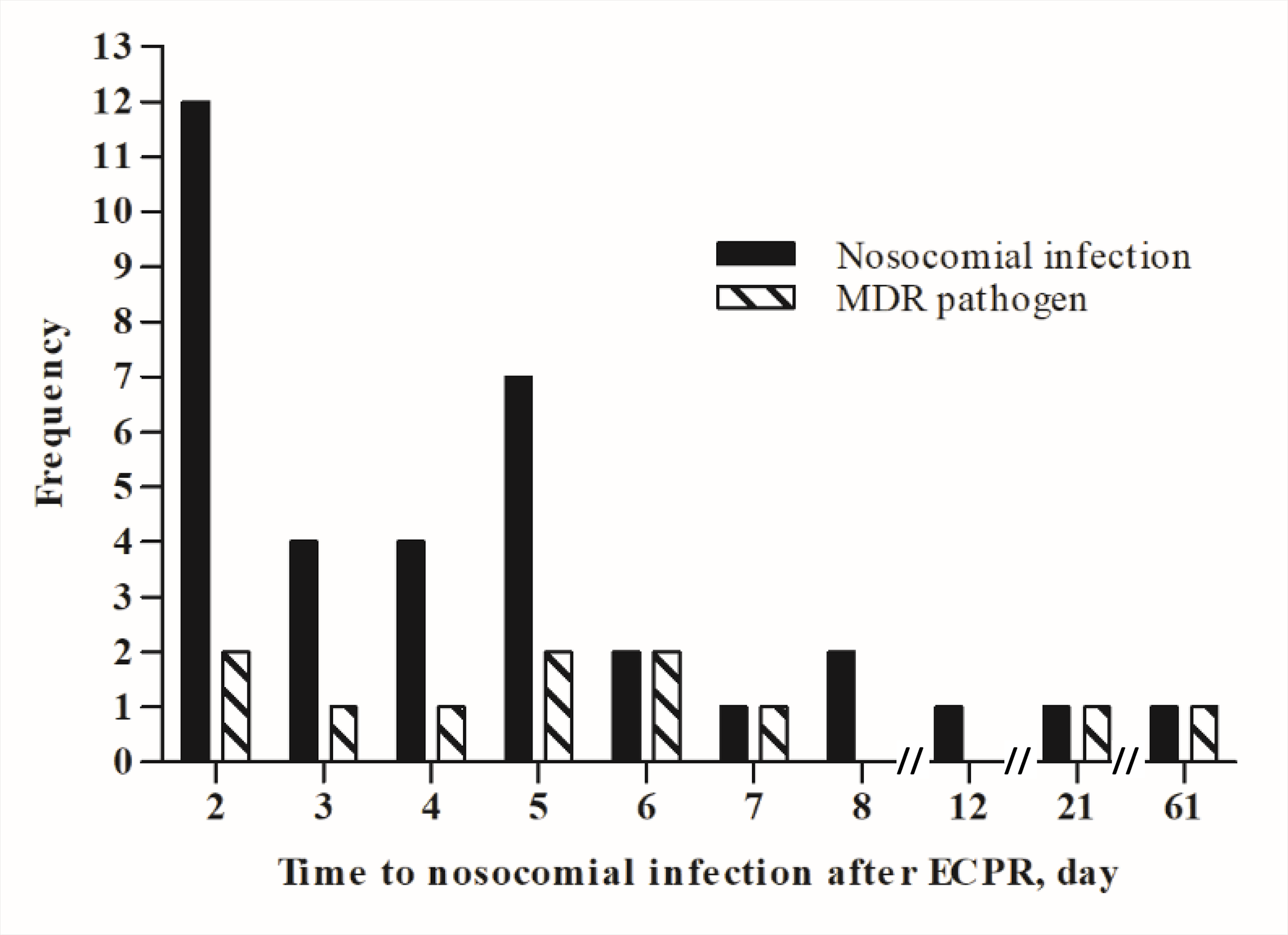

Supplement: S1 Fig — MDR = multidrug resistant, ECPR = extracorporeal cardiopulmonary resuscitation. (TIF) [file pone.0243838.s005.tif]

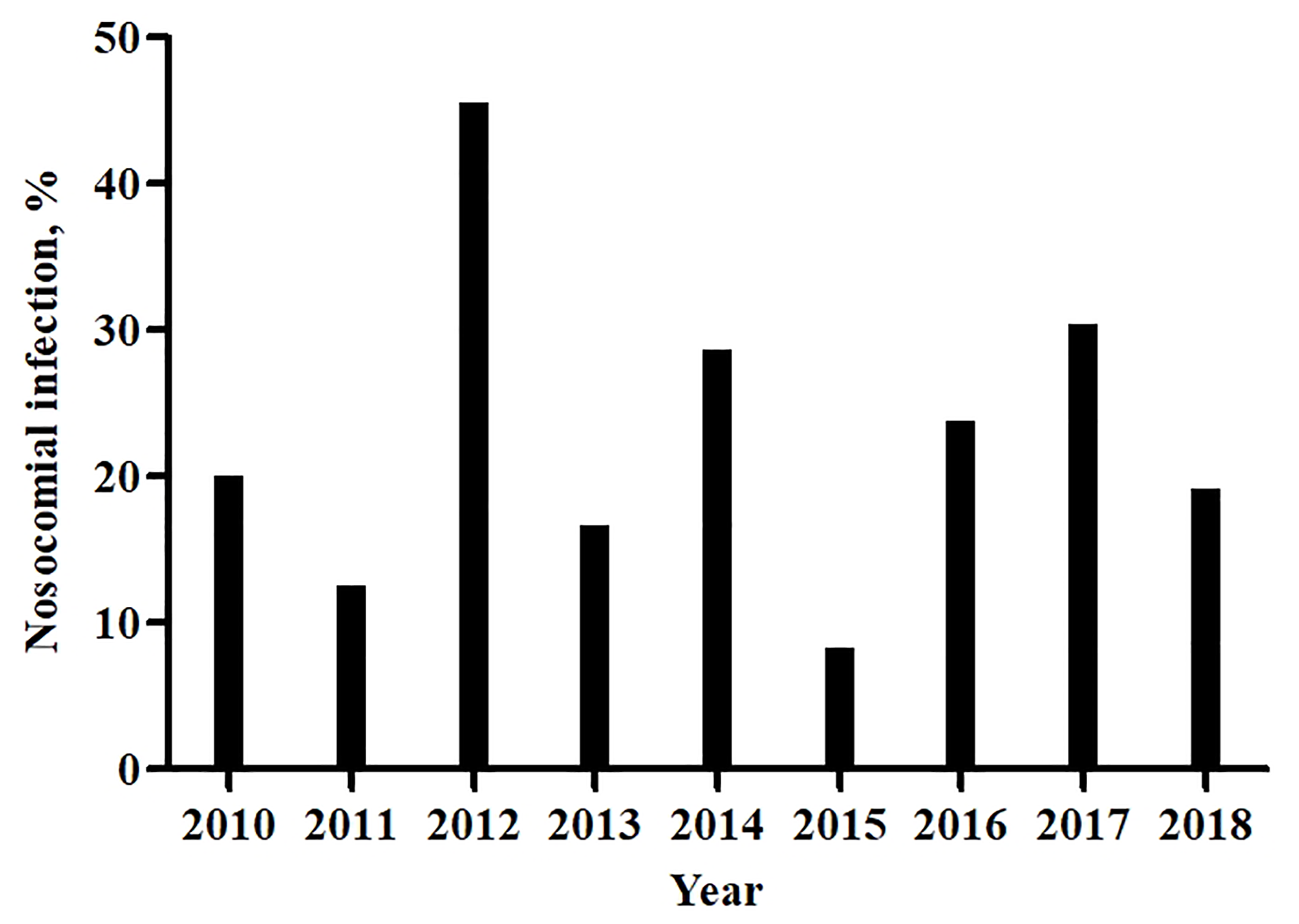

Supplement: S2 Fig — (TIF) [file pone.0243838.s006.tif]
